# Communication during the initial visit to a CPAP clinic Practitioners’ experiences of facilitators and barriers when talking to patients with obstructive sleep apnea

**DOI:** 10.1111/jsr.13244

**Published:** 2020-12-13

**Authors:** Anders Broström, Bengt Fridlund, Bjöörn Fossum, Amir Pakpour, Per Nilsen, Martin Ulander

**Affiliations:** ^1^ Department of Clinical Neurophysiology University Hospital Linköping Sweden; ^2^ Department of Nursing School of Health and Welfare Jönköping University Jönköping Sweden; ^3^ Centre for Interprofessional Collaboration within Emergency care (CICE) Linnaeus University Växjö Sweden; ^4^ Sophiahemmet University Stockholm Sweden; ^5^ Karolinska Institutet Department of Clinical Science and Education Södersjukhuset, Stockholm Sweden; ^6^ Social Determinants of Health Research Center Research Institute for Prevention of Non‐Communicable Diseases Qazvin University of Medical Sciences Qazvin Iran; ^7^ Department of Health, Medicine and Caring Sciences Division of Society and Health Linköping University Linköping Sweden; ^8^ Department of Clinical and Experimental Medicine Division of Clinical Neurophysiology Faculty of Medicine Linköping University Linköping Sweden

**Keywords:** adherence, communication, continuous positive airway pressure, obstructive sleep apnea, shared decision‐making

## Abstract

Adherence to continuous positive airway pressure treatment for obstructive sleep apnea tends to be poor. Communication influences adherence but has not previously been investigated from a practitioner perspective, although shared decision‐making is known to be of great importance. The aim was to describe how practitioners experience communication with patients with obstructive sleep apnea during the initial visit at a continuous positive airway pressure treatment clinic, with focus on facilitators and barriers related to the 4 Habits Model, a communication model comprised of four types of interrelated skills to make encounters more patient‐centred: investing in the beginning; exploring the patient perspective; showing empathy; and investing in the end. A descriptive design with qualitative content analysis was used. A deductive analysis was carried out based on interviews with 24 strategically selected practitioners from seven continuous positive airway pressure treatment clinics. The 4 Habits Model was used as a framework for identifying facilitators and barriers to communication. *Investments in the beginning* was described as creating contact, showing the agenda and being adaptive, while *explore the patient perspective* included showing awareness, being explorative and creating a participating climate. *Show empathy* consisted of showing openness, being confirmative and creating acceptance, while showing a structured follow‐up plan, being open minded and invitational and creating motivation to build on were descriptions of *invest in the end*. Awareness of potential facilitators and barriers for patient‐centred communication during the beginning, middle and end of a continuous positive airway pressure treatment consultation can be used to improve contextual conditions and personal communication competences among practitioners working with continuous positive airway pressure treatment initiation.

## 1 INTRODUCTION

Obstructive sleep apnea (OSA) is a chronic and highly prevalent public health problem in which the soft tissue of the upper airways collapses during sleep, leading to obstructive apneas and hypopneas (Senaratna et al., 2017). This, in turn, may cause sleep fragmentation, sympathetic nervous system activation and episodic oxygen desaturation. Untreated severe OSA is associated to hypertension, cardiovascular morbidity and mortality, diabetes, traffic and occupational accidents, and poor quality of life (Tietjens et al., 2019). Continuous positive airway pressure (CPAP), a device that prevents the airways from collapsing by creating a positive airway pressure during sleep, has been offered to patients with OSA during the last 20 years (Rotenberg et al., 2016). CPAP care is typically provided by practitioners (e.g. nurses or technicians), who are responsible for educating and motivating the patients. Even though the effectiveness of the treatment has increased due to significant improvements of devices and masks (Bakker et al., 2019; Baratta et al., 2018), as well as development of improved educational (Wozniak et al., 2014) and psychological interventions (Crawford et al., 2014), adherence is still considered a clinical problem, especially during the early treatment phase (Mehrtash et al., 2019). The initiation procedure is complex, with practitioners having to cover a wide range of educational topics. Flaws in communication about motivational and behavioural aspects (e.g. attitudes, self‐efficacy, illness and treatment beliefs; Broström et al., 2011; Crawford et al., 2014; Ward et al., 2014), need for self‐care actions, and handling of side‐effects (Ulander et al., 2014) have been reported as important barriers to adherence (Bakker et al., 2019). Involvement in decisions and trust in practitioners may have positive effects on motivation to use CPAP treatment (Broström, Nilsen, et al., 2010). However, a current and widespread problem is limited organizational resources (Broström et al., 2018; Karlsson et al., 2015), which contribute to making the communication process task‐oriented (Broström et al., 2017), and restricting practitioners’ ability to follow the patient's agenda and create a situation characterized by shared decision‐making (Elwyn et al., 2012). Further, excessive daytime sleepiness (i.e. to the extent that some patients may literally fall asleep during consultations) and cognitive dysfunction due to chronic poor sleep (Tietjens et al., 2019) may negatively affect the ability to participate in patient education and shared decision‐making. Facilitators and barriers to achieving patient‐centred communication between practitioners and patients during the initial visit to a CPAP clinic have not been investigated from the perspective of practitioners, despite the potential importance of such communication for shared decision‐making (Charles et al., 1997).

A recent survey described that practitioners perceived knowledge as one of the three main determinants for CPAP adherence (Broström, Pakpour, Nilsen, Gardner, et al., 2018). Opportunities for patients to describe and communicate his/her preferences depend on the communicative space they are given by nurses and the importance given to create a shared treatment decision (Shay & Lafata, 2015). Patient involvement when initiating CPAP ranges from answering simple questions about symptoms to actively participating in decision‐making regarding, for example, mask adaptation or adjusting the settings on the CPAP device (Broström, Nilsen, et al., 2010). A study (Broström et al., 2017) investigated facilitators and barriers for communication, as described by patients with OSA, and found that structure building, information transfer and commitment from practitioners were of importance to build confidence in the beginning of a CPAP consultation. Organizational insufficiency, stressed behaviour or an interaction deficit were described as barriers for confidence building. A communicational disagreement (i.e. structural obscurity, irresponsibility and absent‐mindedness) was described as a barrier. Agreement regarding responsibilities, a confirmation and comprehensive information were by the patients described as facilitators in the end of the CPAP initiation.

The 4 Habits Model (Frankel & Stein, 1999) describes four key habits, i.e. interrelated skills that facilitate a patient‐centred communication, to be used at the beginning, middle and end of a consultation. The model can be used as a structured way to study different habits during a consultation process. It has been used in many contexts (for review, see Frankel & Sherman, 2015) in terms of eliciting the patient's perspective, understanding the patient within his or her own context, reaching a shared understanding of the patient's problem and its treatment, but also in helping the patient share power by offering him or her meaningful involvement in choices relating to his or her health. However, it has not previously been used to analyse how practitioners communicate with patients in CPAP care.

In a CPAP context, the first habit of the model (Frankel & Stein, 1999), i.e. *investing in the beginning*, could mean that the nurse initiates the meeting in a warm and friendly manner so that the patient feels comfortable and at ease. The second habit, i.e. *eliciting the patient's perspective,* could mean that the nurse explores how the patient understands his/her OSA‐related problems and asks for their expectations. The third habit, i.e. *demonstrating empathy*, could mean that the nurse is open to expressions of anxiety, and shows acceptance of worries and concerns related to symptoms and treatment. The fourth habit, i.e. *investing in the end*, could mean that the nurse provides instructions and asks questions to ascertain that the patient has grasped the information and justifies the CPAP treatment without using an overly technical jargon.

Having an understanding of facilitators and barriers to achieving the four habits would potentially enable CPAP nurses to adapt their communication to be more patient‐centred, thus creating favourable conditions for good adherence in CPAP treatment. However, no study has been conducted to investigate communication habits during the initial meeting at a CPAP clinic, as experienced by the nurse. Therefore, we aimed to describe how practitioners experience communication with patients with OSA during the initial visit at a CPAP clinic, with focus on facilitators and barriers related to the 4 Habits Model.

## 2 METHODS

### 2.1 Design and population

A descriptive deductive design with qualitative content analysis (Graneheim & Lundman, 2004) was used and reported according to the COREQ checklist. Data were collected at five hospital‐based CPAP clinics (i.e. two university hospitals and three county hospitals) and two private clinics located in different parts of the country. The seven clinics were selected based on location, size (i.e. number of treatment initiations per week) and initiation routines (i.e. time from diagnosis to start of treatment, staffing, and time per visit), with the aim to be representative and transferable from a national perspective.

The initial visits that were studied were all individual and part of usual care. They took place 1 week up to 6 weeks after the patients had been objectively diagnosed with OSA. CPAP practitioners performed appointments according to the following clinical routine: visits lasted for 30−90 min, and included oral information about OSA/CPAP, practical adaptation of the mask and an explanation of the device. Subsequent re‐visits depended on the outcome of the initiation, but did in most cases include multiple visits during the following 6 months.

A total of 24 informants were strategically selected with the intention to achieve a clinically sound variation of practitioners working in CPAP care based on gender, age, profession, education level, time since graduation, professional experience working with CPAP initiation, and employment at different type of clinics (i.e. University hospital, County hospital, private clinic; Table 1). Ethical approval was obtained from the Regional Ethics Committee in Linköping, Sweden (Dnr 2014/232‐31).

**TABLE 1:** Sociodemographic characteristics of the participants (*N* = 24)

Sex
Male: 6
Female: 18
Age (years)
≤ 35:0
36–45:7
46–55:8
≥ 56:9
Employment
University hospital: 5
County hospitals: 8
Private clinic: 11
Profession
Registered Nurse: 12
Biomedical analyst/technician: 12
Education level
No‐BSc: 10
BSc: 8
MSc: 6
Time since graduation (years)
≤ 5:0
6–10:3
≥ 11:21
Experience in CPAP care (years)
≤ 5:9
6–10:5
≥ 11:10

### 2.2 Data collection

After being informed about the study, the head of each CPAP clinic approved its effectuation. Based on the strategic selection criteria, practitioners at each clinic were informed about the study through an invitational letter sent by a member of the study group. The letter included information that the interviews should focus specifically on experiences of communication during the initial visit, but they were not told about the specific study aim, or that a deductive design based on the 4 Habits Model would be used. All participants gave written informed consent to participate and interviews being audio recorded. Before the interview, the interviewer explained the study in more detail and emphasized the interviewer's intention to explore experiences of communication. The semi‐structured interviews were conducted in a separate room at each clinic by the first author (AB), who did not have any personal relationship with any of the participants. The pilot tested interview guide was based on five questions, as follows. (a) Can you describe situations that affect communication between you and the patient during the initial visit? (b) Can you describe situations at the beginning of the visit that affect communication? (c) Can you describe situations that affect how you explore the patients’ situation? (d) Can you describe situations that affect how you create contact and show empathy during the conversation? (e) Can you describe situations that affect the ending of the conversation? Probing questions, such as: “Can you describe this in more detail?”, “How did it affect the conversation?”, “What did you say next?” and “What were you thinking?” were used. The duration of the audio‐recorded interviews ranged from 15 to 75 min.

### 2.3 Data analysis

Verbatim transcripts of all interviews were produced, resulting in 295 A4 pages of single‐spaced text in 12‐point Times font. The deductive analysis was performed by a multi‐professional team of clinicians and researchers (1 physician, 3 nurses, 1 psychologist and 1 behavioural scientist, and based on the 4 Habits Model; Frankel & Stein, 1999). Firstly, the literally transcribed interviews were read and checked for accuracy by the lead author (a nurse). Secondly, four of the researchers (1 physician and 3 nurses), all with extensive knowledge regarding OSA, CPAP, communication and qualitative content analysis, carefully searched for statements specifically describing experiences of communication. Thirdly, these descriptions were compared and, from a deductive perspective, clustered into relevant communication habits included in the 4 Habits Model (i.e. invest in the beginning, elicit the patient's perspective, demonstrate empathy, and invest in the end). Fourthly, after in‐depth discussions, including all members of the group, facilitators and barriers were identified. Finally, all authors agreed upon a structured hierarchical description associated with quotations of facilitators and barriers for communication according to each habit included in the 4 Habits Model.

## 3 RESULTS

### 3.1 Invest in the beginning

**FIGURE 1:**
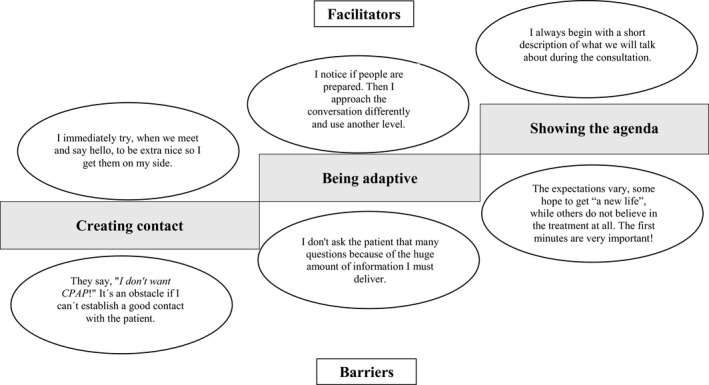
A hierarchical description of facilitators and barriers for investing in the beginning in communication between practitioners and patients during the initial visit to a continuous positive airway pressure (CPAP) clinic

Creating contact, showing the agenda and being adaptive were described as hierarchical investments in the beginning (Figure 1). Facilitators for creating contact included greeting and presenting oneself nicely and correctly in the waiting room, sometimes with the help of jokes, to create a relaxed personal atmosphere based on trust. Barriers were described as meeting patients who directly gave the impression to have forgotten previous information, or showing a negative attitude, low motivation or sometimes even hostility. Facilitators for showing the agenda included that the nurse, when arriving in the room, in an open and friendly way initiated the conversation by describing the agenda (i.e. content and procedures) for the visit in a clear and straightforward way. Barriers then were described as the experience of meeting a patient who had a vague understanding of OSA, its consequences and preposterous expectations of the CPAP treatment, as well as being negative and unappreciative of his or her referral to the clinic. Facilitators for being adaptive were carefully reading the medical record to understand the variety of suitable topics, such as comorbidities or driving‐license related aspects, but also using eye contact, taking in body language and facial expressions, as well as actively looking for signs of knowledge and preunderstanding of OSA and CPAP treatment. Being stressed, not having all the facts about the specific situation, meeting a taciturn patient with an unclear or closed body language, feeling a need to deliver huge amounts of information during short visits, and prioritizing one‐way communication about practical aspects were barriers for being adaptive.

### 3.2 Explore the patient perspective

**FIGURE 2:**
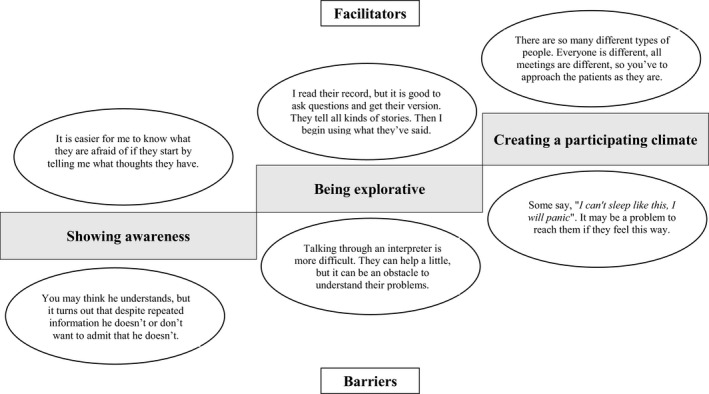
A hierarchical model including facilitators and barriers for exploration of the patient perspective in communication between practitioners and patients during the initial visit to a continuous positive airway pressure (CPAP) clinic

Showing awareness, being explorative and creating a participating climate were described as hierarchical ways to explore the patient perspective (Figure 2). Facilitators for showing awareness included meeting a patient who easily, without the need of frequent probing questions, described the situation, while barriers were described as meeting a patient who hesitated to communicate his or her expectations, or had difficulties to understand the presented information, or showed lack of competence to understand technical device or mask‐related aspects, either due to cognitive, linguistic, psychological or social aspects. Unlimited time, and having an understanding of how to use a holistic patient‐centred approach to guide the communication and explore relevant medical, pathophysiological, anatomical, practical as well as behavioural aspects were described as facilitators for being explorative, while a conversation with low interaction, characterized by few questions and comments from the patient, or language barriers and a need for an interpreter were described as barriers. Factors that facilitated creating a participating climate included being friendly, and giving time and space to the patient to think, but also an understanding of how to adapt one's own communication and behaviour to match different personality traits, while strong emotional reactions, such as anger, fear, anxiety and claustrophobia were described as barriers difficult to tackle.

### 3.3 Show empathy

**FIGURE 3:**
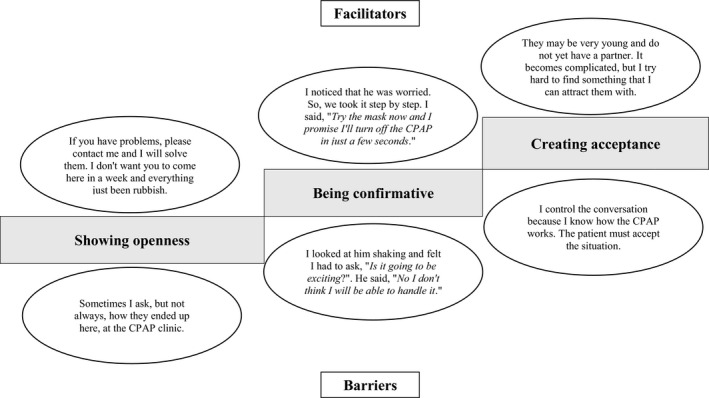
A hierarchical model including facilitators and barriers for showing empathy in communication between practitioners and patients during the initial visit to a continuous positive airway pressure (CPAP) clinic

Showing openness, being confirmative and creating acceptance were described as hierarchical ways to show empathy (Figure 3). Facilitators for showing openness included an invitational patient‐centred communication approach when talking to the patient, a sense of being able to connect based on quick personal responses to open‐ended questions, and making assurances to solve problems. Impressions of the patient being in a bad mood, for example, by use of language or facial expressions, the patient taking a passive role, or avoiding responding to personal questions were described as barriers for showing openness. Carefully listening to descriptions of emotional treatment‐related reactions, such as fear, anxiety, depressive symptoms and crying, as well as responding to them professionally were described as facilitators, while misinterpreting the responses, having difficulties to take in problems mentioned or being misled by preconceived opinions were described as barriers for being confirmative. Facilitators for creating acceptance included confirming reactions, talking in a compassionate way, and trying to calm the patient with explanations or technical demonstrations, sometimes by using humour, as well as offering a positive patient‐ and age‐centred target image, while practitioners who needed to control the conversation by deciding topics being talked about, due to feeling more knowledgeable, and thereby hindering patients to communicate their preferences were described as barriers.

### 3.4 Invest in the end

**FIGURE 4:**
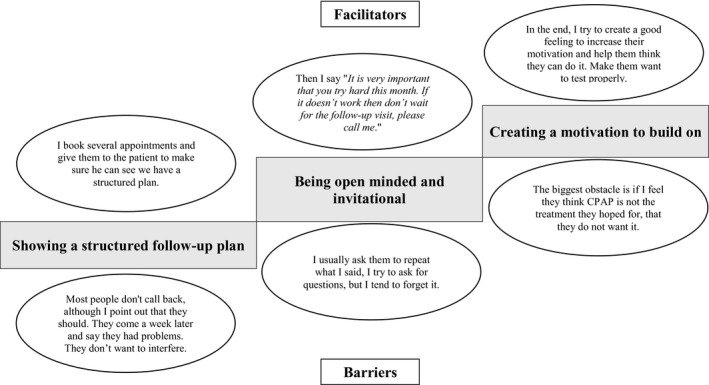
A hierarchical model including facilitators and barriers for investing in the end of consultations between practitioners and patients during the initial visit to a continuous positive airway pressure (CPAP) clinic

Showing a structured follow‐up plan, being open minded and invitational, and creating motivation to build on were described as hierarchical ways to invest in the end (Figure 4). Facilitators for showing a structured follow‐up plan were: presenting a several‐appointment schedule, based on national guidelines, but individually adapted to the patient needs, and clearly communicate the potential but flexible agenda for the scheduled appointments, including expectations, without creating stress, while patients who gave the feeling of not wanting to interfere were described as barriers. Asking if there were topics not covered and the patients understanding of theoretical as well as practical aspects, examine the need for repetition of practical mask‐ and device‐related procedures, and clearly highlighting the importance of using contact details to get in touch if problems arose were described as facilitators, while forgetting to ask about the patients understanding, or if they had questions, were barriers for being open minded and invitational. Facilitators for creating motivation to build on included use of positively expressed sentences to try hard, mentioning of positive both physical and psychosocial treatment outcomes intended to create hope and a good vibe, but threats about risks of apneas, desaturations and living with untreated OSA were also used, while having a clear feeling that the patient did not want the CPAP, by expressing a negative attitude, arguing for other options were described as barriers.

## 4 DISCUSSION

We found that the first step of the 4 Habits Model, investing in the beginning, was described as creating contact, showing the agenda and being adaptive, while the second step, exploring the patient perspective, involved showing awareness, being explorative and creating a participating climate. The third step, showing empathy, consisted of showing openness, being confirmative and creating acceptance, while the fourth step, investing in the end, involved showing a structured follow‐up plan, being open minded and invitational and creating motivation to build on. We identified a wide range of facilitators and barriers based on these aspects in all steps of the model. These facilitators and barriers have not been described from a practitioner perspective before, despite their potential importance for achieving improved CPAP adherence.

Initiation of CPAP is a complex and often lengthy process, involving practitioners from different clinics. Continuity of care, workflow characteristics of the healthcare setting and time are aspects that can either facilitate or hinder shared decision‐making (Joseph‐Williams et al., 2014). During the CPAP initiation there are conversations with the patient about diagnostic procedures, diagnosis, treatment options, and treatment decisions by physicians and practitioners (Epstein et al., 2009). Various theoretical and practical CPAP‐related topics are communicated, often spanning a period of several months with long periods of waiting in‐between visits. The process typically begins in primary care, with symptom screening being carried out by a primary care physician before the patient is referred to a sleep clinic, where diagnostic‐ and registration‐specific areas are communicated by physicians, before a practitioner meets the patient to initiate CPAP treatment. Eliciting a good understanding of the patient's perspective, including descriptions of the whole life situation and reaching shared treatment decisions based on the patient's values, is highly important when creating a patient‐centred communication approach when initiating CPAP treatment (Thériault & Grad, 2019). However, even if psychological interventions have been highlighted as important for CPAP adherence (Crawford et al., 2014), it has also been shown that patients expect the communication during the initial visit to be focused on disease and biomedical issues (Broström et al., 2017), which might be attributed to traditions and the complex process involving numerous professionals. Still, pathophysiology, results from the diagnostic procedure and aspects related to the technical device must be addressed by the practitioner during a relatively short visit (Broström, Nilsen, et al., 2010). Hence, even if the practitioner (e.g. a nurse, biomedical analyst or technician) has a relevant understanding of determinants of adherence and desires to use a patient‐centred communication approach, it can be difficult to ensure shared treatment‐related decisions focusing on the patient's perspective (Broström, Pakpour, Nilsen, Gardner, et al., 2018). Different professions also have different educational backgrounds and belong to different professional cultures, which might affect their communication approach and how topics might be emphasized. A communication technique based on the 4 Habits Model, accounting for the facilitators and barriers identified in the current study, might therefore provide a means for CPAP practitioners of different professions to increase patient‐centred communication and reduce the biomedical focus and medical jargon in attempts to persuade or even threaten the patient to achieve long‐term CPAP adherence (Broström et al., 2017).

Practitioners used positive, friendly comments about non‐medical problems or even humour to build rapport during the first contact at the beginning of the visit. This finding is in line with Hashim’s (2017) list of phrases to be used during the introduction phase to establish favourable conditions for a good communication situation in a medical setting. However, it is worth noting that if a consultation begins with a phrase based on an open‐ended question it may encourage the patient to begin talking about physical symptoms. If instead the practitioner uses an open‐ended question that is focused on the patient's perspective of CPAP (Ward et al., 2014), for example, feelings or concerns related to the initiation of CPAP, the focus of the conversation is more likely to be non‐medical. Initial open‐ended explorative questions could according to Hashim’s (2017) recommendation be “How do you feel about the CPAP treatment?” or “What do you worry about regarding the CPAP treatment?” A strategy could therefore be to, after the initial phrases, begin by asking several questions concerning feelings, concerns and expectations of the actual CPAP treatment, and then turn to negotiate the topics, and ask the patient to prioritize which question is the most important due to the restricted time. The patient's primary concerns should be explored. Preferably, the conversation should cover both patient preferences and issues of medical importance. Conversations about less prioritized concerns might be postponed to follow‐up visits if they are not considered to affect the initial CPAP use (Broström, Pakpour, Nilsen, Gardner, et al., 2018), thus allowing more focus on the following steps in the 4 Habits Model.

Showing awareness, being explorative and creating a participating climate were ways practitioners used to explore the patient perspective. The patient's perception of his or her illness is the primary focus of patient‐centred care (Dwamena et al., 2012). Asking about his or her understanding of OSA and treatment needs may provide an understanding of how to increase treatment motivation (Drieschner et al., 2004). Exploring the patient's feelings is important in assessing the emotional burden and psychological impact of the illness to promote autonomy (Epstein & Street, 2011). Fears related to patient's own perceptions of symptoms or to descriptions delivered by partners (e.g. fear of apneas or claustrophobia) may require a thorough exploration and probing questions of the patient's preunderstanding (Broström, Johansson, et al., 2007), which may help the practitioner to prioritize and address communicational topics of importance.

Some previous studies (Broström, Nilsen, et al., 2010; Broström, Pakpour, Nilsen, Gardner, et al., 2018) have shown that practitioners sometimes use fear of symptoms or bring up consequences of OSA, for example, apneas, desaturations and cardiovascular disease, as motivational aspects to achieve CPAP treatment adherence. In terms of the Self‐Determination Theory (Deci & Ryan, 1985), emphasizing negative aspects of OSA to achieve CPAP treatment adherence involves introjected motivation and/or externally regulated motivation, neither of which is a type of motivation that yields stable and sustainable behaviours performed with care and quality (Ryan & Deci, 2000). Important aspects to consider are expectations, needs, intentions and desires related to the treatment in general (Broström et al., 2017), fears for being prescribed a CPAP mask (Broström, Nilsen, et al., 2010), but also how personality (Broström, Strömberg, et al., 2007) as well as physical and cognitive states (Leng et al., 2017) may affect the conversation. Epstein and Street (2011) mean that a situation in which a practitioner gets to understand the patient's perspective of a situation is based on shared knowledge and shared deliberations through sharing thoughts, feelings, perceptions, meanings and intentions with the patient. This can be done either verbally (i.e. with open‐ended questions, continuers, legitimation, understanding, exploration, rephrasing or checking the patient's understanding) or non‐verbally (i.e. by using different aspects of body language, such as: attention, responsiveness, attentiveness, openness, interest, active listening or focus; Hashim, 2017). Interpersonal sensitivity (Hall, 2011) is a crucial ability to accurately read and understand other people's feelings and states, and to respond appropriately, which are important social interaction skills when exploring the OSA patient's perspective. Training for practitioners could be offered to improve the aforementioned competences.

A combination of linguistic exactness and empathy, in which practitioners provide the patient with direct, neutral and evidence‐based information, but also confirm emotions and thoughts, shows respect and provides support, contributing to creating a shared decision (Callon et al., 2018). We found that practitioners used a structured follow‐up plan, tried to be open minded and invitational, and create motivation to build on in the end of the visit. Openness, confirmation and acceptance were ways to express empathy. However, when entering the last phase of the visit, even if important, empathic motivational talk should be carefully considered. Empathy is the capacity to understand and relate to how the patient with OSA experiences his/her illness and emotions, but also empathy for his/her expressions of treatment expectations. Importantly, empathy could be communicated through exploring experiences and emotions related to the patient's OSA, such as limiting symptoms of fatigue or sleepiness, or showing understanding, respect and support for claustrophobia related to the CPAP mask (Broström et al., 2017). Hashim (2017) states that there are different techniques for expressing empathy to patients. In a CPAP context this could be exemplified as naming (e.g. “It seems like you are worried about using a CPAP mask”), understanding (e.g. “I understand that your sleepiness must be difficult to cope with”), respecting (e.g. “I’m impressed by your motivation”), supporting (e.g. “Promise to call me if you have any problems with your mask”) or exploring (e.g. “Do you have any questions about the CPAP device?”). A practitioner who explores the patient perspective with relevant questions, and by showing awareness, being explorative, as well as by creating a participating climate may, based on his/her expressed empathy, have a better chance to develop an adherent CPAP behaviour. Fostering favourable conditions for internal motivation to build on could improve adherence, and using balanced arguments based on a risk−benefit analysis is highly important. However, it is important to consider that the patient's capacity could be affected due to cognitive problems caused either by severe symptoms (Broström, Johansson, et al., 2007) or disease‐related consequences. This was confirmed by the findings in the present study, for example, when practitioners described cognitive aspects as a barrier for exploring the patient perspective. Multiple sessions may be required to achieve behavioural change and adherent CPAP use considering age and e‐health literacy as barriers and the substantial amount of information needed in relation to the complexity of the condition, diagnostic procedures, technical and practical aspects of the treatment (Broström et al., 2014). Practitioners in the present study expressed using a combination of obtaining positive treatment effects and the risk of suffering cardiovascular disease if OSA was untreated as motivational factors. This has been found as a strategy also in other studies (Broström, Pakpour, Nilsen, Gardner, et al., 2018), but could be a considerable risk if the patient cannot use the CPAP as prescribed.

In clinical practice, patient‐centred communication could be complemented by brief instruments measuring attitude (Broström et al., 2011) and motivation (Broström et al., 2020). The instruments could be sent to the patient before the initial visit to adapt OSA/CPAP education and optimize the practitioner approach to the situation during the initial visit. Two other instruments (i.e. SURE and Collaborate) could be used after the initial visit to screen for decisional conflict and to evaluate shared decision‐making (Broström, Pakpour, Nilsen, Gardner, et al., 2018). Instruments for side‐effects (Broström, Franzen Arestedt et al., 2010) and CPAP habit development (Broström et al., 2014) could be used to target, adapt and evaluate practitioners’ interventions during the second and third follow‐up visits (i.e. after several weeks of treatment), as side‐effects tend to vary and patients respond differently to interventions, which might affect habit development (Ulander et al., 2014). The same instruments complemented with, for instance, Calgary Sleep Apnea Quality of Life Index (Flemons & Reimer, 2002) and Ethos Brief Index (Broström et al., 2019) might be applied to evaluate mediators and moderators for CPAP adherence, but also to establish whether patient‐centred communication during clinical visits facilitates the patient's adherence to CPAP, or whether a specific therapeutic orientation (e.g. psychological) is required.

### 4.1 Methodological considerations

There are some methodological aspects to be considered regarding trustworthiness (i.e. credibility, dependability, confirmability and transferability). Credibility was based on the connection between the aim and the chosen method, i.e. qualitative content analysis (Graneheim & Lundman, 2004). However, another qualitative method, for example, critical incident technique, describing both experiences and actions in relation to decisive situations, might have added other aspects to the findings. Profession and professional cultures might affect communication approach, but the strategically selected informants, 12 nurses and 12 biomedical analysts/technicians showed a relevant variation in terms of occupation in comparison to what is seen in clinical practice. Credibility was also enhanced by the multidisciplinary research team as it allowed different perspectives on the issue under study. Dependability was strengthened by a piloted interview guide used by an experienced interviewer who was aware of his preunderstanding and added relevant probing questions to elaborate experiences described during interviews. Moreover, all collected data were carefully checked for accuracy by a researcher with an understanding of both the context and methodological aspects. The deductive analytical process was based on the 4 Habits Model, and involved repeated discussions and reflections by a multi‐professional team with long clinical experience of CPAP treatment as well as analytical experience of using qualitative content analysis. This deductive approach might have limited the variation of the findings, and there may also be a difficulty in distinguishing between the different habits. Confirmability was strengthened by the hierarchical description of facilitators and barriers being associated with quotations. Transferability was based on the variation in the clinical and sociodemographic characteristics of the practitioners from a variety of clinics in different parts of the country and the professions who are involved in CPAP initiation (i.e. Registered Nurses, Biomedical analysts and technicians).

## 5 CONCLUSION

Creating contact, showing the agenda and being adaptive were used in the beginning of conversation, while showing awareness, being explorative and creating a participating climate were used to explore the patient perspective. Showing empathy consisted of showing openness, being confirmative and creating acceptance, while showing a structured follow‐up plan, being open minded and invitational and creating motivation to build on were descriptions of investing in the end.

## Conflict of interest

None.

## Data Availability

The data that support the findings of this study are available from the corresponding author upon reasonable request.
